# Inflammatory biomarkers show sex-specific associations with cardiometabolic disease and coronary atherosclerosis as assessed by coronary CT angiography

**DOI:** 10.1016/j.ajpc.2026.101718

**Published:** 2026-06-26

**Authors:** Daniel O. Bittner, Caroline Roesner, Dorette Raaz-Schrauder, Markus Goeller, Damini Dey, Tobias Kilian, Stephan Achenbach, Mohamed Marwan

**Affiliations:** a Friedrich-Alexander University Erlangen-Nürnberg (FAU), Department of Cardiology, Erlangen, Germany; b University Hospital Augsburg, Department of Cardiology, Augsburg University, Germany; c Biomedical Imaging Research Institute, Cedars-Sinai Medical Center, Los Angeles, CA, USA

**Keywords:** Cardiometabolic disease, Inflammatory cytokines, Inflammatory biomarkers, Coronary atherosclerosis, Coronary artery disease, Sex differences, Coronary CT angiography

**Figure:**
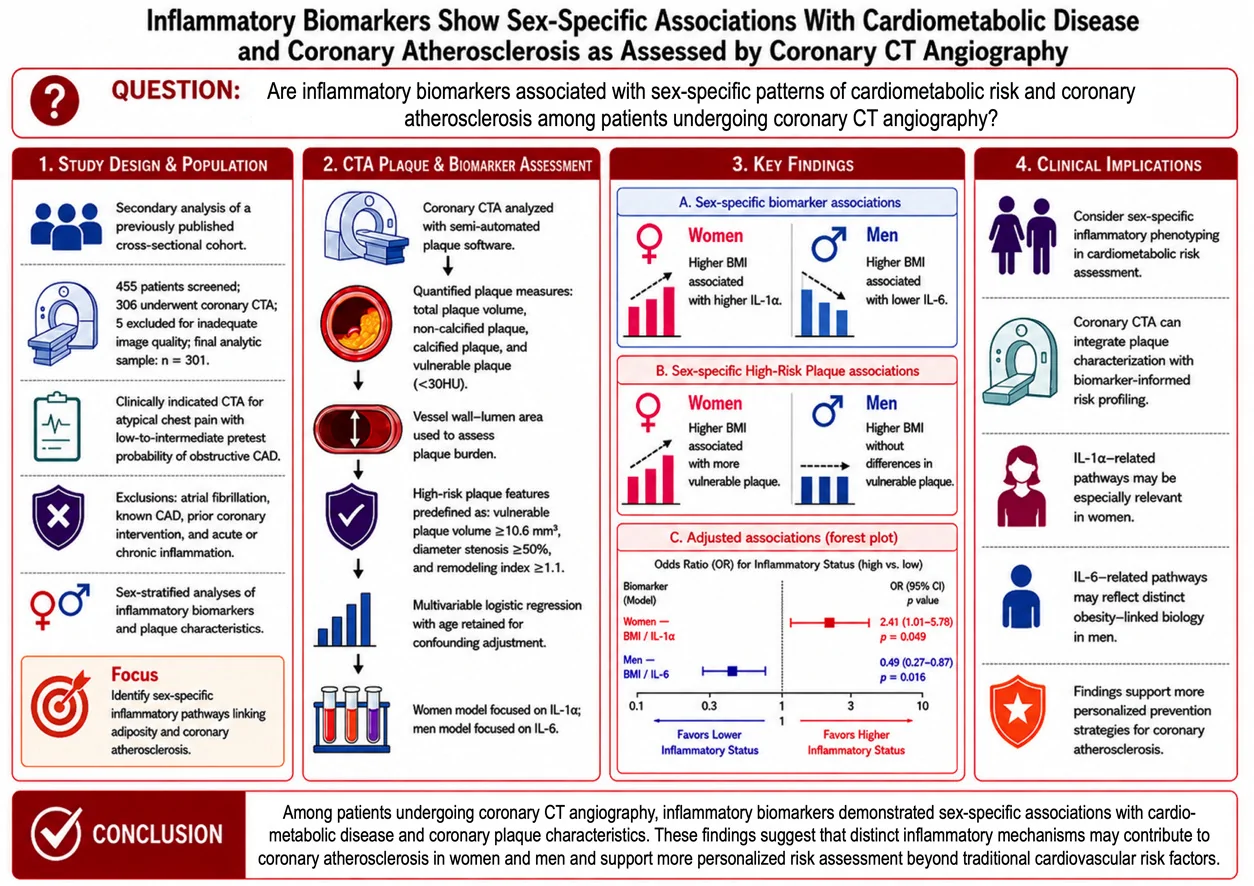

## Background and aims

Metabolic dysregulation and chronic inflammation are central drivers of cardiometabolic disease [1]. Adipose tissue–derived cytokines, including IL-6 and IL-1 family members, contribute to systemic inflammation and coronary atherosclerosis [2] yet their sex-specific relevance remains incompletely understood despite known differences in inflammatory and cardiometabolic phenotypes between men and women [3].

Coronary CT angiography (CTA) enables non-invasive detection of coronary artery disease (CAD) and provides detailed characterization of coronary plaque morphology and vulnerability.

To address current knowledge gaps we investigated whether higher body mass index (BMI) is associated with sex-specific inflammatory cytokine profiles and relates to the extent and morphology of CAD as assessed by coronary CTA.

## Methods

### Study design and population

This secondary analysis of a previously published cross-sectional cohort (n = 455)[4] included patients undergoing coronary CTA (n = 306), with 5 excluded for inadequate image quality (final n = 301). CTA was clinically indicated for atypical chest pain with low-to-intermediate pretest probability of obstructive CAD. Exclusion criteria comprised atrial fibrillation, known CAD, prior coronary interventions, and evidence of acute or chronic inflammation. All participants provided written informed consent, and the study was approved by the institutional review board (FAU Erlangen/Nürnberg).

### Coronary plaque analysis

Coronary atherosclerosis was assessed quantitatively and qualitatively using semi-automated software (Autoplaque, Version 2.5; Cedars-Sinai Medical Center, Los Angeles, CA, USA), with scan-specific threshold–based algorithms [5]. Coronary segments with a luminal diameter >2 mm were included for quantification of total (mm³), non-calcified, calcified and low-attenuation plaque volume (= vulnerable plaque) with CT attenuation <30 Hounsfield units (HU).

The semi-automated workflow comprised vessel extraction, identification of proximal and distal plaque boundaries, automated (with optional manual refinement) delineation of the outer vessel wall and contrast-enhanced lumen with plaque thresholding, and calculation of the vessel wall–lumen area as a measure of plaque burden.

Predefined dichotomized high-risk plaque features (HRP) were: vulnerable plaque volume ≥10.6mm³ with CT attenuation <30 HU [6], diameter stenosis (DS) ≥50% and remodeling index (RI) ≥1.1.

### Serum cytokine levels

Serum biomarkers were quantified from venous blood samples obtained at the time of the CT examination. A Luminex-based multiplex assay was used to measure concentrations (pg/mL) of IL-1a, IL-1β, IL-2, IL-4, IL-6, IL-7, IL-8, IL-10, IL-12, IL-13, IL-15, IL-17, interferon-γ, TNF-α, high-sensitivity C-reactive protein (hsCRP), GM-CSF, G-CSF, MCP-1, MIP-1α, and eotaxin [4].

### Statistical analysis

Continuous variables are presented as mean ± SD or median (IQR), and categorical variables as counts (%); correlations were assessed by Spearman’s rho, categorical comparisons by Pearson’s χ² test, and nonparametric continuous variables by Kruskal–Wallis tests across BMI tertiles. To account for multiple testing, Bonferroni correction was included. In multivariable logistic regression analyses, IL-1a (women) and IL-6 (men) were modeled as outcomes, with covariates showing significant univariate associations and age retained to account for potential confounding. A two-sided p-value <0.05 was considered significant using IBM SPSS Statistics, Version 29.0.1.0.

## Results

Out of 301 patients, 94 (31%) were female and 207 (69%) were male. Women and men were stratified according to BMI tertiles in low, medium and high as listed in Tables A and B.

**Tables A and B:** BMI-associated differences across tertiles in women (Panel A) and men (Panel B) in respect to traditional cardiovascular risk factors, coronary artery plaque characteristics and serum cytokine levels.

	BMI			
Baseline Characteristics	Low	Medium	High	
FEMALE	n = 32	n = 31	n = 31	
	*n (%) or mean (±SD)*			p-value
Age (y)	60 (±10)	63 (±9)	63 (±8)	0.28
BMI (kg/m2)	22 (±2)	26 (±1)	31 (±4)	**<0.01**
Cardiovascular riskfactors (CVRF)				
Diabetes mellitus	0 (0%)	5 (16%)	4 (13%)	0.07
Hyperlipidemia	13 (41%)	21 (68%)	22 (71%)	**0.03**
Hypertension	17 (53%)	25 (81%)	25 (81%)	**0.02**
Current or past tabacco use	13 (41%)	10 (32%)	8 (26%)	0.46
Family history of CAD	15 (47%)	8 (26%)	13 (42%)	0.20
Number of CVRF	1,8 (±1,2)	2,2(±1,0)	2,3(±1,3)	0.21

**Coronary Plaque Analysis**					
		*median (IQR)*			p-value
Total plaque volume	mm³	127 (0; 657)	370 (0; 934)	289 (90; 796)	0.23
Total NCP volume	mm³	127 (0; 630)	370 (0; 920)	286 (90; 743)	0.22
Total CP volume	mm³	0 (0; 1)	0 (0; 12)	0 (0; 5)	0.09
Vulnerable plaque volume	mm³	1 (0; 52)	29 (0; 126)	38 (7; 157)	0.07
Diameter stenosis	%	24 (0; 40)	25 (0; 43)	32 (9; 44)	0.34
Remodeling index	%	1.1 (0.0; 1.3)	1.2 (0.0; 1.4)	1.3 (1.0; 1.5)	0.39
		*n (%) or mean (±SD)*			
Vulnerable plaque volume	< 10.6 mm³	19 (59%)	14 (45%)	9 (29%)	**0.05**
	≥ 10.6 mm³	13 (41%)	17 (55%)	22 (71%)	
Remodeling Index	< 1.1	16 (50%)	13 (42%)	13 (42%)	0.76
	≥1.1	16 (50%)	18 (58%)	18 (58%)	
Diameter stenosis	< 50%	30 (94%)	25 (81%)	28 (90%)	0.25
	≥ 50%	2 (6%)	6 (19%)	3 (10%)	
Number of HRP features		1.0 (±1.0)	1.3 (±1.1)	1.4 (±1.0)	0.22

**Serum Cytokine Levels**				
	*median (IQR)*			p-value
IL_1a	3.6 (3.2; 20.2)	13.9 (3.2; 112.0)	25.0 (12.4; 641)	**<0.01**

**Baseline Characteristics**	**BMI**			
	**Low**	**Medium**	**High**	
**MALE**	n = 71	n = 70	n = 66	

	*n (%) or mean (±SD)*			p-value
Age (y)	58 (±9)	57 (±10)	57 (±10)	0.85
BMI (kg/m2)	24 (±2)	27 (±1)	32 (±3)	**<0.01**
Cardiovascular riskfactors (CVRF)				
Diabetes mellitus	5 (7%)	3 (4%)	11 (17%)	**0.03**
Hyperlipidemia	45 (63%)	42 (60%)	43 (65%)	0.82
Hypertension	35 (49%)	43 (61%)	46 (70%)	**0.05**
Current or past tabacco use	32 (45%)	33 (47%)	41 (62%)	0.10
Family history of CAD	26 (37%)	27 (39%)	27 (41%)	0.88
Number of CVRF	2.0 (±1.0)	2.1 (±1.0)	2.6(±1.2)	**<0.01**

**Coronary Plaque Analysis**					
		*median (IQR)*			p-value
Total plaque volume	mm³	465 (170; 942)	407 (155; 946)	506 (247; 882)	0.77
Total NCP volume	mm³	460 (169; 912)	405 (152; 885)	456 (229; 832)	0.80
Total CP volume	mm³	5 (0; 44)	3 (0; 27)	4 (0; 31)	0.68
Vulnerable plaque volume	mm³	33 (5; 171)	24 (5; 95)	48 (6; 143)	0.36
Diameter stenosis	%	35 (19; 53)	37 (26; 46)	35 (27; 44)	0.87
Remodeling index	%	1.2 (1.1; 1.4)	1.2 (1.1; 1.4)	1.2 (1.1; 1.4)	0.95
		*n (%) or mean (±SD)*			
Vulnerable plaque volume	< 10.6 mm³	28 (39%)	28 (40%)	21 (32%)	0.55
	≥ 10.6 mm³	43 (61%)	42 (60%)	45 (68%)	
Remodeling Index	< 1.1	22 (31%)	21 (30%)	18 (27%)	0.89
	≥1.1	49 (69%)	49 (70%)	48 (73%)	
Diameter stenosis	< 50%	52 (73%)	58 (83%)	57 (86%)	0.13
	≥ 50%	19 (27%)	12 (17%)	9 (14%)	
Number of HRP features		1.6 (±1.1)	1.5 (±1.0)	1.5 (±0.9)	0.86

**Serum Cytokine Levels**				
	*median (IQR)*			p-value
IL_6	9.7 (3.2; 27.6)	6.5 (3.2; 24.9)	3.3 (3.2; 12.7)	**0.01**
IL_10	11.8 (3.2; 34.0)	19.8 (3.8; 41.1)	7.3 (3.2; 16.1)	**0.02**
MIP_1a	14.5 (3.2; 40.6)	19.6 (3.2; 36.9)	7.8 (3.2; 19.3)	**0.02**

IQR: interquartile range; NCP volume: non-calcified plaque volume; CP volume: calcified plaque volume; HRP: high-risk-plaque features (defined as presence of vulnerable plaque volume ≥ 10.6 mm³, remodeling index ≥1.1 and/or diameter stenosis ≥50%).

### Cardiovascular risk profiles and BMI

In women, BMI positively correlated only with hyperlipidemia (0.299; p < 0.01) and hypertension (0.300; p < 0.01) with prevalence increasing across BMI tertiles (all p < 0.05). In univariate analysis BMI showed significant association to hyperlipidemia (OR 3.09; 95%CI 1.31–7.27; p = 0.010) and hypertension (OR 3.15; 95%CI 1.23–8.07; p = 0.017), but not number of CVRF (p = 0.53).

In men, BMI positively correlated with hypertension (0.170; p = 0.02), diabetes (0.134; p = 0.05), current or prior smoking (0.157; p = 0.02) and number of CVRF (0.231; p < 0.001). Across BMI tertiles, the prevalence of diabetes, hypertension and number of CVRF increased (p < 0.01). In univariate analysis BMI showed significant association to hypertension (OR 1.84; 95%CI 1.05–3.24; p = 0.034), but not diabetes or number of CVRF (p > 0.05).

### Coronary plaque burden/morphology and BMI

BMI in women positively correlated with vulnerable plaque volume (0.255; p = 0.01) and threshold ≥10.6mm³ (0.257; p = 0.01).Vulnerable plaque volume showed an increase from 1 to 38 mm³ across BMI tertiles, missing significance (p = 0.07) and in univariate analysis (OR 2.02; 95%CI 0.89–4.61; p = 0.094). However, the prevalence of vulnerable plaque volume threshold ≥10.6mm³ increased from 41% to 71% across BMI tertiles (p = 0.05) with positive association (OR 2.55; 95%CI 1.10–5.89; p = 0.028). The increase in mean HRP features (1.0 to 1.4) and prevalence of ≥1 HRP feature (56% to 77%) was not significant (all p > 0.05; regression analysis: OR 2.16 (95%CI 0.91–5.16; p = 0.082)).

BMI in men showed neither correlation to plaque measures (all p > 0.05) nor differences across BMI tertiles (all p > 0.05).

### Inflammatory biomarkers and BMI

BMI in women positively correlated only with IL-1a (0.314; p < 0.01), increasing from 3,6 to 25,0 pg/ml across BMI tertiles (p < 0.01), with significant association in univariate analysis (OR 2.82; 95%CI 1.22–6.53; p = 0.015). In multivariable analysis, treating IL1a as outcome, only BMI showed significant association with IL-1a (OR 2.41; 95%CI: 1.01–5.78; p = 0.049), independent of hyperlipidemia, hypertension and age (all p > 0.05).

In men, BMI inversely correlated with IL-13 (−0.175; p = 0.01), IL-1a (−0.190; p > 0.01), IL-1b (−0.138; p = 0.05), IL-6 (−0.178; p = 0.01) and IL-8 (−0.157; p = 0.02). IL-10 and MIP-1a showed an inverted U-shaped pattern, IL-6 a linear decrease (9,7 to 3,3 pg/ml) across BMI tertiles (p < 0.05). In univariate analysis, only IL-6 showed a significant inverse association to BMI (OR 0.53; 95%CI 0.31–0.93; p = 0.026). In multivariable analysis, treating IL-6 as outcome, BMI showed a significant inverse association (OR 0.49; 95%CI: 0.27–0.87; p = 0.016) with IL-6, independent of hypertension (OR 1.99; 95%CI: 1.07–3.68; p = 0.029) and age and diabetes (both p > 0.05).

## Discussion

In our analysis, traditional cardiovascular risk factors increased with BMI in both sexes, whereas a trend toward higher vulnerable plaque burden was observed only in women. Increasing IL-1a levels in women and decreasing IL-6 levels in men with higher BMI further suggest sex-specific inflammatory pathways contributing to coronary atherosclerosis.

In this post-hoc analysis, traditional cardiovascular risk factors increased with BMI in both sexes, whereas coronary plaque differences were not uniformly observed, suggesting that conventional risk factors alone may not fully explain atherogenesis. Men with lower BMI and fewer risk factors showed plaque burden comparable to men with higher BMI, while the inverse association between BMI and IL-6 suggests a complex, potentially sex-specific interplay between adiposity, inflammation, and atherosclerosis. While adipose tissue represents a relevant source of circulating IL-6, previous studies have yielded heterogeneous findings regarding the association between obesity and IL-6, particularly in sex-stratified analyses [[7], [8]]. Accordingly, the present findings should be interpreted with caution and viewed as exploratory, without permitting causal or mechanistic conclusions regarding adipose tissue–driven inflammation.

IL-1 family cytokines are central mediators of vascular inflammation and plaque destabilization, yet IL-1a remains less extensively studied than IL-6 or CRP. In our cohort, IL-1a was positively associated with BMI and showed a trend toward increased vulnerable plaque volume exclusively in women, suggesting potential sex-specific inflammatory pathways, although interpretation is limited by the number of female participants. Given the established pro-inflammatory role of IL-1a, these findings may support IL-1–targeted therapeutic strategies in cardiovascular disease. Previous trials investigating IL-1 inhibition with anakinra demonstrated attenuation of inflammatory biomarkers in acute coronary syndromes, although long-term clinical benefits remain inconsistent [[9], [10]]. Whether women with higher BMI derive particular benefit from IL-1 inhibition warrants evaluation in larger prospective studies.

### Limitations

These associations derive from post-hoc analyses of a cross-sectional cohort at a single time point and should be considered hypothesis-generating; establishing causality will require large-scale, longitudinal randomized controlled trials. Incomplete baseline pharmacotherapy data represent a limitation, as statins and ACE inhibitors exert anti-inflammatory and plaque-stabilizing effects beyond their primary cardiovascular actions. These pleiotropic effects may confound the observed associations by directly modulating systemic inflammation and atherosclerotic biology. Although individuals with overt inflammatory conditions were excluded, subclinical inflammation may have remained undetected as high-sensitivity C-reactive protein measurements were obtained exclusively for research purposes. BMI was used as an indirect surrogate of adiposity; however, the lack of measures of central adiposity such as waist circumference or visceral fat quantification limits mechanistic interpretation regarding adipose tissue–driven inflammation.

## Conclusion

Despite a higher prevalence of traditional cardiovascular risk factors with increasing BMI, a trend to higher vulnerable plaque volume were observed only in women. Sex-specific BMI-associated cytokine profiles suggest inflammatory pathways contributing to coronary atherosclerosis beyond traditional risk factors.

Conflict of interest.

The authors declare that there is no conflict of interest related to this work.

## Declaration of generative AI and AI-assisted technologies in the manuscript preparation process

During the preparation of this work, the author(s) used ChatGPT for the creation of the central illustration. The author(s) reviewed and edited the output as needed and take full responsibility for the content of the published article.

## CRediT authorship contribution statement

**Daniel O. Bittner:** Conceptualization, Methodology, Validation, Formal analysis, Investigation, Resources, Data curation, Writing – original draft, Writing – review & editing, Supervision, Project administration. **Caroline Roesner:** Conceptualization, Methodology, Validation, Formal analysis, Investigation, Resources, Data curation, Writing – original draft, Writing – review & editing, Visualization, Project administration. **Dorette Raaz-Schrauder:** Resources, Data curation, Writing – review & editing. **Markus Goeller:** Resources, Writing – review & editing. **Damini Dey:** Resources, Writing – review & editing. **Tobias Kilian:** Resources, Writing – review & editing. **Stephan Achenbach:** Writing – review & editing, Supervision, Project administration. **Mohamed Marwan:** Writing – review & editing, Supervision, Project administration.

## Declaration of competing interest

The authors declare that they have no known competing financial interests or personal relationships that could have appeared to influence the work reported in this paper.

The author is an Editorial Board Member/Editor-in-Chief/Associate Editor/Guest Editor for this journal and was not involved in the editorial review or the decision to publish this article.

The authors declare the following financial interests/personal relationships which may be considered as potential competing interests:

Dr. Dey reports royalties from Cedars-Sinai Medical Center, outside the submitted work; In addition, Dr. Dey has a patent US8885905B2 in U.S. and WO patent WO2011069120A1. Dr. Marwan reports honoraria from Siemens HealthCare and Edwards Lifescience outside the submitted work. All other authors have nothing to disclose. All other authors have nothing to disclose.
